# Hospital Accreditation Status and Treatment Differences Among Black Patients With Colon Cancer

**DOI:** 10.1001/jamanetworkopen.2024.29563

**Published:** 2024-08-21

**Authors:** Kelley Chan, Bryan E. Palis, Joseph H. Cotler, Lauren M. Janczewski, Ronald J. Weigel, David J. Bentrem, Clifford Y. Ko

**Affiliations:** 1 American College of Surgeons Cancer Programs, Chicago, Illinois; 2 Department of Surgery, Stritch School of Medicine, Loyola University Chicago, Maywood, Illinois; 3 Department of Surgery, Northwestern University Feinberg School of Medicine, Chicago, Illinois; 4 Department of Surgery, Carver College of Medicine, The University of Iowa, Iowa City; 5 Department of Surgery, David Geffen School of Medicine at UCLA, Los Angeles, California

## Abstract

**Importance:**

Hospital-level factors, such as hospital type or volume, have been demonstrated to play a role in treatment disparities for Black patients with cancer. However, data evaluating the association of hospital accreditation status with differences in treatment among Black patients with cancer are lacking.

**Objective:**

To evaluate the association of Commission on Cancer (CoC) hospital accreditation status with receipt of guideline-concordant care and mortality among non-Hispanic Black patients with colon cancer.

**Design, Setting, and Participants:**

This population-based cohort study used the National Program of Cancer Registries, which is a multicenter database with data from all 50 states and the District of Columbia, and covers 97% of the cancer population in the US. The participants included non-Hispanic Black patients aged 18 years or older diagnosed with colon cancer between January 1, 2018, and December 31, 2020. Race and ethnicity were abstracted from medical records as recorded by health care facilities and practitioners. The data were analyzed from December 7, 2023, to January 17, 2024.

**Exposure:**

CoC hospital accreditation.

**Main Outcome and Measures:**

Guideline-concordant care was defined as adequate lymphadenectomy during surgery for patients with stages I to III disease or chemotherapy administration for patients with stage III disease. Multivariable logistic regression models investigated associations with receipt of guideline-concordant care and Cox proportional hazards regression models assessed associations with 3-year cancer-specific mortality.

**Results:**

Of 17 249 non-Hispanic Black patients with colon cancer (mean [SD] age, 64.8 [12.8] years; 8724 females [50.6%]), 12 756 (74.0%; mean [SD] age, 64.7 [12.8] years) were treated at a CoC-accredited hospital and 4493 (26.0%; mean [SD] age, 65.1 [12.5] years) at a non–CoC-accredited hospital. Patients treated at CoC-accredited hospitals compared with those treated at non–CoC-accredited hospitals had higher odds of receiving guideline-concordant lymphadenectomy (adjusted odds ratio [AOR], 1.89; 95% CI, 1.69-2.11) and chemotherapy (AOR, 2.31; 95% CI, 1.97-2.72). Treatment at CoC-accredited hospitals was associated with lower cancer-specific mortality for patients with stages I to III disease who received surgery (adjusted hazard ratio [AHR], 0.87; 95% CI, 0.76-0.98) and for patients with stage III disease eligible for chemotherapy (AHR, 0.75; 95% CI, 0.59-0.96).

**Conclusions and Relevance:**

In this cohort study of non-Hispanic Black patients with colon cancer, patients treated at CoC-accredited hospitals compared with those treated at non–CoC-accredited hospitals were more likely to receive guideline-concordant care and have lower mortality risk. These findings suggest that increasing access to high-quality guideline-concordant care at CoC-accredited hospitals may reduce variations in cancer treatment and outcomes for underserved populations.

## Key Points

**Question:**

Is Commission on Cancer (CoC) hospital accreditation status associated with receipt of guideline-concordant care and risk of mortality among Black patients with colon cancer?

**Findings:**

In this cohort study of 17 249 non-Hispanic Black patients with colon cancer, treatment at a CoC-accredited hospital was associated with higher odds of receiving guideline-concordant care and lower risk of cancer-specific mortality.

**Meaning:**

The findings of this study indicate that increasing access to CoC-accredited hospitals and upholding requirements for benchmarking adherence to guideline-concordant care may represent opportunities to reduce variations in cancer treatment and outcomes for underserved populations.

## Teaser

This cohort study uses the National Program of Cancer Registries database to evaluate the association between Commission on Cancer accreditation status of hospitals and receipt of guideline-concordant care and risk of mortality among non-Hispanic Black patients with colon cancer.

## Introduction

Despite overall increases in the availability of screening modalities and improvements in the treatment of colon cancer, disparities persist in the treatment and outcomes of colon cancer for underserved populations.^1^ Black patients have a higher incidence of colon cancer, are more often diagnosed at advanced stages, are less likely to receive guideline-concordant care (GCC), and have worse overall outcomes compared with White patients with colon cancer.^2,3^ Disparities in access to high-quality cancer care have been attributed to several factors, including geographic and sociodemographic characteristics.^4^

Benchmarking adherence to GCC may represent an opportunity to minimize variation in treatment and improve disparities in treatment and outcomes for patients with colon cancer. The receipt of GCC has been associated with improved survival for patients with colon cancer.^5,6^ However, disparities have persisted in the adoption of high-quality cancer treatment guidelines for Black patients compared with White patients across multiple cancer sites.^7,8^ Hospital accreditation has been associated with multiple domains of health care quality including safety, timeliness, effectiveness, efficiency, and patient centeredness.^9^ The American College of Surgeons (ACS) was the first standard setting organization to develop criteria for hospital accreditation in 1919 to improve the care of surgical patients.^10^ The ACS Commission on Cancer (CoC) was established in 1922 to create standards to ensure high quality, multidisciplinary, and comprehensive cancer care delivery.^11^ Currently, the CoC assesses the quality of approximately 1400 CoC-accredited hospital cancer programs in the US. Through hospital accreditation, the CoC aims to establish and maintain evidence-based standards of care, evaluate hospital performance against these standards, and promote quality assessment and improvement.^11^

Studies have suggested that hospital-level factors, including type and volume, may play a role in cancer treatment disparities among Black patients compared with those in White patients.^12,13^ However, data evaluating the association of hospital accreditation status with differences in treatment among Black patients with cancer are lacking. The objective of this study was to evaluate the association of CoC hospital accreditation status with receipt of GCC and risk of mortality among non-Hispanic Black (hereafter, Black) patients with colon cancer.

## Methods

### Data Source

In this cohort study, the United States Cancer Statistics database, which includes cancer registry data from the Centers for Disease Control and Prevention National Program of Cancer Registries (NPCR) and the National Cancer Institute (NCI) Surveillance, Epidemiology, and End Results (SEER) Program database, was queried to identify patients diagnosed with colon cancer between January 1, 2018, and December 31, 2020.^14^ The United States Cancer Statistics database captures data on patient demographic characteristics, tumor characteristics, and treatment from all 50 states and the District of Columbia and covers 97% of the cancer population.^15^ This study was conducted in compliance with the institutional review board protocol of the American College of Surgeons, with informed consent requirements waived because the data within the NPCR and SEER Program database were deidentified. This retrospective cohort study followed the Strengthening the Reporting of Observational Studies in Epidemiology (STROBE) reporting guideline.^16^

### Study Population

Black patients aged 18 years or older with newly diagnosed colon cancer with epithelial tumor histology were included. Colon cancer was identified using the *International Classification of Diseases for Oncology*, *Third Revision*, topography codes C18.0 and C18.2-C18.9.^17,18^ All cases were abstracted according to the instructions and coding definitions of the North American Association of Central Cancer Registries.^19^ Cases were included from reporting hospitals identified as non–CoC-accredited or CoC-accredited at the time of case abstraction. Analytic cases prepared at CoC-accredited hospitals included cases where all or part of the first course treatment was performed at the reporting facility. Cases were excluded from the study if abstracted from a death certificate, abstracted from a non-hospital source, if none of the first course of treatment was given at the reporting hospital, if the first course of treatment was unknown, or if the case was nonanalytic. Cases were excluded if disease stage, insurance type, or Census tract poverty indicator was missing. Given that patients with cancer are frequently treated at multiple facilities, a majority of central cancer registries use probabilistic record linkage with public and private health insurance claims to extract treatment information.^20,21,22^

### Variables

The following patient-level variables were evaluated across the entire cohort: age at diagnosis (years), sex, insurance type, rural or urban location, geographic location, and US Census tract poverty indicator. Race and ethnicity were abstracted from medical records as recorded by health care facilities and practitioners. The only hospital-level characteristic available in the NPCR database was the CoC accreditation status at the time the case was abstracted. Tumor-level characteristics included primary site, pathological grade, and disease stage, which was determined by the NCI SEER Summary Stage converted to the American Joint Committee on Cancer Staging Manual, 8th edition.^23,24,25^ Stages I to III included patients with SEER Summary Stages of “localized only,” “regional by direct extension only,” and “regional lymph node involvement.” Stage III included patients with the SEER Summary Stage of “regional lymph node involvement.” Outcome variables included cancer-specific mortality and survival months.

### Outcomes and Definitions

The primary outcomes of the study were receipt of GCC and risk of mortality. Eligibility and receipt of GCC were defined according to the National Comprehensive Cancer Network treatment guidelines.^26^ Guideline-concordant lymphadenectomy was defined as a minimum of 12 lymph nodes examined for patients with stages I to III disease who received surgery defined as partial colectomy, subtotal colectomy or hemicolectomy, total colectomy, total proctocolectomy, and colectomy, not otherwise specified. Patients were considered ineligible for guideline-concordant lymphadenectomy if they had evidence of metastatic disease, did not receive surgery, refused surgery, or if surgery was contraindicated due to patient risk factors. Guideline-concordant chemotherapy was defined as administration of single or multiple agent chemotherapy or chemotherapy, not otherwise specified, for patients with stage III disease. Patients were considered ineligible for guideline-concordant chemotherapy if they had local or metastatic disease, died prior to chemotherapy, refused chemotherapy, if chemotherapy was contraindicated due to patient risk factors, or if their age was older than 80 years. The mortality outcome included an evaluation of 3-year cancer-specific mortality, which was based on the number of months between the date of diagnosis and the date of death or December 31, 2021, whichever came first. State cancer registries are linked to the National Center for Health Statistics’ National Death Index. Registrars use a predefined algorithm based on causes of death from death certificates, determined by the *International Statistical Classification of Diseases and Related Health Problems, Tenth Revision, Clinical Modification* codes, to establish cancer-specific mortality based on tumor sequence, site of original cancer diagnosis, and comorbidities.^27^ Cancer-specific survival obtained using NPCR data for colorectal cancer has 95% detection and confirmation rates.^28^

### Statistical Analysis

We used χ^2^ test statistics to compare categorical study population characteristics for patients evaluated at CoC-accredited and non–CoC-accredited hospitals and *t* test statistics to compare differences in percent adherence with the lymphadenectomy and chemotherapy measures by hospital accreditation status. Multivariable logistic regression models were performed to investigate factors associated with receipt of guideline-concordant lymphadenectomy during surgery and guideline-concordant chemotherapy, adjusting for patient sociodemographic characteristics, primary site, stage, and hospital accreditation. Three-year survival estimates were obtained from Kaplan-Meier survival curves, using the log-rank and Wilcoxon rank sum tests for patients diagnosed in 2018 having at least 36 months of accrued follow-up time. Multivariable risk-adjusted 3-year cancer-specific mortality hazard ratios were calculated with fixed-effects Cox proportional hazards regression models for both treatment-eligible cohorts, adjusting for patient sociodemographic characteristics, primary site, stage, and hospital accreditation. Additionally, a sensitivity analysis was performed for receipt of GCC of Black patients with low socioeconomic status (SES), defined as residing in a Census tract with 20% to 100% poverty, to evaluate whether treatment concordance was associated with SES. Analyses were conducted from December 7, 2023, to January 17, 2024, using SAS, version 9.4 (SAS Institute Inc). The threshold for statistical significance was set at *P* < .05 (2-tailed).

## Results

Of the 17 249 Black patients (mean [SD] age, 64.8 [12.8] years; 8724 females [50.6%]; 8525 males [49.4%]) included in this study, 12 756 (74.0%) were evaluated at a CoC-accredited hospital and 4493 (26.0%) at a non–CoC-accredited hospital (Table 1, Figure). Among the Black patients evaluated at CoC-accredited hospitals, a higher proportion of patients resided in metropolitan locations, lived in the Southeast and Northeast geographic regions, and were diagnosed with higher-stage disease. Compared with Black patients evaluated at CoC-accredited hospitals, a higher proportion of those evaluated at non–CoC-accredited hospitals resided in urban and rural locations, lived in the South geographic region, and lived below the poverty level. There was no significant difference in age at diagnosis or insurance type between patients evaluated at a CoC-accredited hospital and those at a non–CoC-accredited hospital. Additionally, there was no significant difference in the proportion of patients who received surgery at a CoC-accredited hospital compared with those treated at a non–CoC-accredited hospital (96.9% vs 96.4%, *P* = .08).

**Table 1.** Sociodemographic and Tumor Characteristics of Non-Hispanic Black Patients Diagnosed With Colon Cancer

Variable	No. (%)		*P* value
	Non–CoC-accredited	CoC-accredited	
Total patients	4493 (26.0)	12 756 (74.0)	
Age, y			
Mean (SD)	65.1 (12.5)	64.7 (12.8)	.12
18-49	443 (9.9)	1432 (11.2)	
50-59	1029 (22.9)	2852 (22.4)	
60-69	1397 (31.1)	3897 (30.6)	
70-79	1012 (22.5)	2910 (22.8)	
≥80	612 (13.6)	1665 (13.1)	
Sex			
Male	2240 (49.9)	6285 (49.3)	.50
Female	2253 (50.1)	6471 (50.7)	
Geographic location			
North	115 (2.6)	557 (4.4)	<.001
Northeast	770 (17.1)	3218 (25.2)	
Midwest	591 (13.2)	2372 (18.6)	
South	1745 (38.8)	2738 (21.5)	
Southeast	1167 (26.0)	3717 (29.1)	
West	105 (2.3)	154 (1.2)	
Insurance type			
Uninsured	196 (4.4)	552 (4.3)	.38
Private	1416 (31.5)	4041 (31.7)	
Medicaid	561 (12.5)	1457 (11.4)	
Medicare	2223 (49.5)	6445 (50.5)	
Other government^a^	97 (2.2)	261 (2.0)	
Rural or urban			
Metropolitan	3543 (78.9)	11 564 (90.7)	<.001
Urban	857 (19.1)	1087 (8.5)	
Rural	93 (2.1)	105 (0.8)	
Census tract poverty indicator			
0% to <5% Poverty	326 (7.3)	1071 (8.4)	<.001
5% to <10% Poverty	612 (13.6)	2182 (17.1)	
10% to <20% Poverty	1295 (28.8)	3830 (30.0)	
20%-100% Poverty	2260 (50.3)	5673 (44.5)	
Primary site			
Right colon	2247 (50.0)	6499 (50.9)	.13
Transverse colon	491 (10.9)	1443 (11.3)	
Left colon	1599 (35.6)	4449 (34.9)	
Overlapping lesion of colon and colon not otherwise specified	156 (3.5)	365 (2.9)	
Grade, pathologic			
Well differentiated	568 (12.6)	1299 (10.2)	<.001
Moderately differentiated	2997 (66.7)	8837 (69.3)	
Poorly differentiated	429 (9.5)	1504 (11.8)	
Undifferentiated	9 (0.2)	22 (0.2)	
GX, unknown	490 (10.9)	1094 (8.6)	
Disease stage			
Localized only	2049 (45.6)	5268 (41.3)	<.001
Regional by direct extension only	867 (19.3)	2542 (19.9)	
Regional lymph node involvement	1577 (35.1)	4946 (38.8)	
Received surgery^b^			
Yes	4073 (96.4)	11 851 (96.9)	.08
No	153 (3.6)	376 (3.1)	

Abbreviations: CoC, Commission on Cancer; GX, grade cannot be assessed.

^a^ Other government insurance included TRICARE, military, Veterans Affairs, and Indian Health Service.

^b^ Patients with unknown surgery status were excluded.

**Figure.**
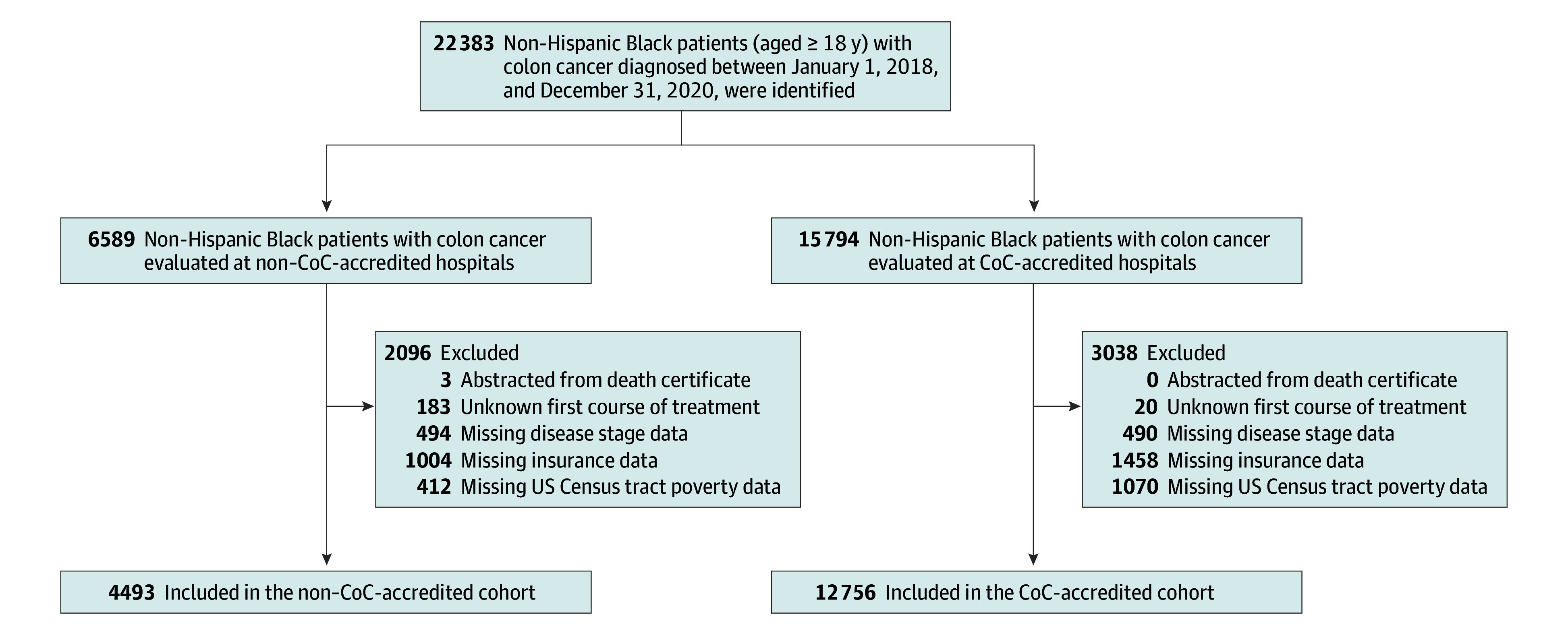
Flow Diagram for the Commission on Cancer (CoC) Accredited and Non–CoC-Accredited Cohorts

Black patients were more likely to receive guideline-concordant lymphadenectomy when treated at CoC-accredited hospitals compared with non–CoC-accredited hospitals (91.9% vs 84.5%, *P* < .001) (Table 2). Black patients were also more likely to receive guideline-concordant chemotherapy when treated at CoC-accredited hospitals compared with non–CoC-accredited hospitals (84.4% vs 70.1%, *P* < .001).

**Table 2.** Comparison of Adherence With Colon Cancer Lymphadenectomy and Chemotherapy Measures

	Eligible patients, No.	Adherent, No. (%)	*P* value
**Lymphadenectomy measure**			
CoC-accredited	11 811	10 852 (91.9)	<.001
Non–CoC-accredited	4047	3420 (84.5)	
**Chemotherapy measure**			
CoC-accredited	3718	3138 (84.4)	<.001
Non–CoC-accredited	1133	794 (70.1)	

Abbreviation: CoC, Commission on Cancer.

Multivariable logistic regression model results indicated that treatment at a CoC-accredited hospital was the factor with the highest adjusted odds of receipt of GCC for Black patients for both treatment measures (Table 3). For patients receiving treatment at a CoC-accredited hospital, compared with those treated at a non–CoC-accredited hospital, the adjusted odds ratios (AORs) of receipt of adequate guideline-concordant lymphadenectomy during surgery was 1.89 (95% CI, 1.69-2.11) and guideline-concordant chemotherapy was 2.31 (95% CI, 1.97-2.72). Patients of female sex compared with male sex had higher odds of receiving GCC. Older age (50 years or older) was associated with lower odds of receiving GCC. Census tract poverty level was not associated with receipt of GCC in either treatment-eligible cohort. Patients with private insurance compared with Medicaid had higher odds of receiving guideline-concordant chemotherapy (AOR, 1.47; 95% CI, 1.14-1.88).

**Table 3.** Multivariable Adjusted Odds Ratio for Receipt of Colon Cancer Lymphadenectomy and Chemotherapy Measures

Variable	Receipt of adequate lymphadenectomy during surgery, AOR (95% CI)	*P* value	Receipt of chemotherapy for stage III disease, AOR (95% CI)^a^	*P* value
Sex				
Male	1 [Reference]	NA	1 [Reference]	NA
Female	1.19 (1.07-1.33)	.001	1.22 (1.05-1.41)	.01
Age, y				
18-49	1 [Reference]	NA	1 [Reference]	NA
50-59	0.78 (0.62-0.98)	.049	0.68 (0.52-0.90)	.005
60-69	0.69 (0.55-0.86)	.03	0.47 (0.35-0.62)	.008
70-79	0.68 (0.53-0.87)	.01	0.29 (0.21-0.39)	<.001
≥80	0.64 (0.49-0.83)	.02	NA	NA
Insurance type				
Medicare	0.76 (0.62-0.92)	.002	1.03 (0.80-1.32)	.62
Private	0.99 (0.81-1.20)	.47	1.47 (1.14-1.88)	<.001
Medicaid	1 [Reference]	NA	1 [Reference]	NA
Uninsured	0.98 (0.71-1.35)	.70	0.83 (0.58-1.21)	.07
Other government^b^	0.99 (0.64-1.53)	.76	1.13 (0.65-1.94)	.81
Rural or urban				
Metropolitan	1.81 (1.57-2.09)	<.001	0.92 (0.73-1.16)	.98
Urban	1 [Reference]	NA	1 [Reference]	NA
Rural	0.97 (0.65-1.46)	.10	0.84 (0.44-1.60)	.67
Census tract poverty indicator				
0% to <5% Poverty	1 [Reference]	NA	1 [Reference]	NA
5% to <10% Poverty	0.88 (0.68-1.13)	.83	0.94 (0.68-1.29)	.67
10% to <20% Poverty	0.82 (0.65-1.04)	.34	1.03 (0.76-1.38)	.40
20%-100% Poverty	0.78 (0.62-0.97)	.02	0.92 (0.69-1.22)	.39
Hospital accreditation				
Non–CoC-accredited	1 [Reference]	NA	1 [Reference]	NA
CoC-accredited	1.89 (1.69-2.11)	<.001	2.31 (1.97-2.72)	<.001
Disease stage^a^				
Localized only	1 [Reference]	NA	NA	NA
Regional by direct extension only	1.67 (1.45-1.93)	.006	NA	NA
Regional lymph node involvement	1.90 (1.69-2.15)	<.001	NA	NA
Primary site				
Right colon	1 [Reference]	NA	1 [Reference]	NA
Transverse colon	0.47 (0.40-0.56)	<.001	0.97 (0.75-1.25)	.67
Left colon	0.44 (0.39-0.49)	<.001	0.86 (0.73-1.01)	.36
Overlapping lesion of colon and colon not otherwise specified	0.68 (0.49-0.94)	.04	0.87 (0.58-1.31)	.72

Abbreviations: AOR, adjusted odds ratio; CoC, Commission on Cancer.

^a^ Only patients with stage III colon cancer were eligible for chemotherapy.

^b^ Other government insurance included TRICARE, military, Veterans Affairs, Indian Health Service.

The 3-year cancer-specific survival for patients with stages I to III disease who received surgery was 82.3% for patients treated at CoC-accredited hospitals and 80.0% for those treated at non–CoC-accredited hospitals (log-rank *P* = .07, Wilcoxon *P* = .03). The 3-year cancer-specific survival for patients with stage III disease eligible for chemotherapy was 77.4% for patients treated at CoC-accredited hospitals and 71.7% for those treated at non–CoC-accredited hospitals (log-rank *P* = .048, Wilcoxon *P* = .02). After adjustment, treatment at CoC-accredited hospitals compared with treatment at non–CoC-accredited hospitals was associated with a lower risk of 3-year cancer-specific mortality for Black patients with stages I to III disease who received surgery (adjusted hazard ratio [AHR], 0.87; 95% CI, 0.76-0.98) and Black patients with stage III disease eligible for chemotherapy (AHR, 0.75; 95% CI, 0.59-0.96) (Table 4). For both treatment-eligible cohorts, older age (60 years and older) compared to younger age (59 years and younger) was associated with a higher risk of mortality. Census tract poverty level was not associated with mortality in either treatment-eligible cohort. Medicare or private insurance compared with Medicaid was associated with lower risk of mortality for both treatment-eligible cohorts.

**Table 4.** Adjusted Cancer-Specific Mortality Hazard Ratios for Non-Hispanic Black Patients Diagnosed in 2018 Eligible for the Lymphadenectomy and Chemotherapy Measures

Variable	Patients who received surgery, AHR (95% CI)	*P* value	Patients eligible for chemotherapy, AHR (95% CI)^a^	*P* value
Sex				
Male	1 [Reference]	NA	1 [Reference]	NA
Female	0.67 (0.60-0.76)	<.001	0.71 (0.58-0.88)	.002
Age, y				
18-49	1 [Reference]	NA	1 [Reference]	NA
50-59	1.29 (0.97-1.72)	.08	1.35 (0.92-2.00)	.13
60-69	1.63 (1.23-2.16)	.001	1.60 (1.08-2.35)	.02
70-79	2.36 (1.75-3.18)	<.001	2.30 (1.49-3.55)	<.001
≥80	4.56 (3.38-6.16)	<.001	NA	
Insurance type				
Medicare	0.81 (0.66-0.99)	.04	0.62 (0.45-0.87)	.005
Private	0.61 (0.49-0.75)	<.001	0.54 (0.39-0.75)	<.001
Medicaid	1 [Reference]	NA	1 [Reference]	NA
Uninsured	0.77 (0.55-1.09)	.14	0.84 (0.52-1.37)	.49
Other government^b^	0.58 (0.35-0.95)	.03	0.42 (0.15-1.16)	.09
Rural or urban				
Metropolitan	0.86 (0.72-1.03)	.10	1.08 (0.77-1.53)	.65
Urban	1 [Reference]	NA	1 [Reference]	NA
Rural	1.29 (0.78-2.13)	.33	0.62 (0.15-2.59)	.51
Census tract poverty indicator				
0% to <5% Poverty	1 [Reference]	NA	1 [Reference]	NA
5% to <10% Poverty	1.20 (0.90-1.58)	.21	1.53 (0.92-2.54)	.10
10% to <20% Poverty	1.16 (0.90-1.50)	.26	1.38 (0.86-2.23)	.01
20%-100% Poverty	1.54 (1.20-1.97)	<.001	1.79 (1.13-2.83)	.01
Hospital accreditation				
Non–CoC-accredited	1 [Reference]	NA	1 [Reference]	NA
CoC-accredited	0.87 (0.76-0.98)	.03	0.75 (0.59-0.96)	.02
Disease stage^a^				
Localized only	1 [Reference]	NA	NA	NA
Regional by direct extension only	1.40 (1.19-1.66)	<.001	NA	NA
Regional lymph node involvement	2.01 (1.75-2.30)	<.001	NA	NA
Primary site				
Right colon	1 [Reference]	NA	1 [Reference]	NA
Transverse colon	1.11 (0.92-1.35)	.27	0.98 (0.69-1.39)	.92
Left colon	1.05 (0.91-1.20)	.52	0.78 (0.61-1.00)	.05
Overlapping lesion of colon and colon not otherwise specified	1.42 (1.05-1.91)	.02	1.10 (0.68-1.77)	.70

Abbreviations: AHR, adjusted hazards ratio; CoC, Commission on Cancer.

^a^ Only patients with stage III colon cancer were eligible for chemotherapy.

^b^ Other government insurance included TRICARE, military, Veterans Affairs, Indian Health Service.

Sensitivity analyses evaluating receipt of GCC among Black patients with low SES likewise showed that treatment at CoC-accredited hospitals remained associated with higher odds of receiving guideline-concordant lymphadenectomy (AOR, 1.95; 95% CI, 1.67-2.29) and chemotherapy (AOR, 2.23; 95% CI, 1.77-2.80) (eTable in Supplement 1).

## Discussion

This cohort study evaluated the association of CoC accreditation status with receipt of GCC and risk of mortality among Black patients with colon cancer. Although there was no difference in receipt of surgery for patients evaluated at CoC-accredited hospitals compared with those treated at non–CoC-accredited hospitals, treatment at a CoC-accredited hospital was associated with higher odds of receiving guideline-concordant lymphadenectomy or chemotherapy and with lower cancer-specific mortality risk.

The results of this study corroborate prior studies^29,30^ analyzing the association of CoC accreditation with GCC in single state cancer registries. While prior studies used single state cancer registries, the results of this study are more nationally representative. For example, a study using the South Carolina Central Cancer Registry to explore racial disparities in the quality of breast cancer treatment reported that CoC-accredited hospitals performed significantly better than non–CoC-accredited hospitals with national quality measures related to the administration of chemotherapy and hormone therapy.^29^ Similarly, a study linking the California Cancer Registry and California Neighborhoods Data System found that Black women with breast cancer who were treated at CoC-accredited hospitals had a survival advantage compared with Black women treated at non–CoC-accredited hospitals.^30^ Both studies highlight key components of CoC accreditation that may have contributed to increased adherence with quality measures and improved survival, including data reporting and feedback of quality measure performance.

Hospital accreditation encompasses the principle that adherence to evidence-based metrics may minimize variations in treatment stemming from nonmodifiable factors. This study found that after adjusting for CoC accreditation status, Census tract poverty level was not associated with receipt of GCC or cancer-specific survival. Among patients with Medicare, differences in GCC predominantly accounted for differences in overall and cancer-specific survival between Black and White patients.^7^ The authors^7^ suggest that while SES may influence mortality, increasing access to GCC is a targetable goal to improve disparities in cancer outcomes. A population-based study demonstrated that all Black patients with colorectal cancer compared with non-Hispanic White patients had an increased risk of death regardless of their Census tract poverty level quintile.^31^ These findings demonstrate the need for further investigation of the influence and interactions of measures of disparity among diverse populations with cancer.

Increasing access to CoC accreditation, which requires benchmarking adherence to GCC, may represent an opportunity to minimize variation in treatment and improve disparities in treatment and outcomes for patients with colon cancer. Among community hospitals, where patients are less likely to receive GCC, participation in a hospital network has been associated with increased compliance with quality measures and attainment of CoC accreditation.^32^ Tucker et al^32^ noted that a key driver for improvements in compliance with quality measures was related to the network requirement for affiliate community hospitals to pursue CoC accreditation, which requires hospitals to implement changes when compliance decreases to below expected levels. A study evaluating changes in compliance with quality measures at community cancer programs after implementation of the CoC Rapid Quality Reporting System (a real-time data evaluation system) reported sustained improvements in the quality of breast and colon cancer care.^33^ However, although overall quality was improved, not all patients in that study benefited equally. These findings support the need for increased focus on initiatives to improve care for diverse populations and the incorporation of equity into standards of cancer care.

In addition to implementing standards to increase the delivery of GCC, CoC accreditation requires hospitals to hold multidisciplinary cancer case conferences and address multiple structural barriers that span the continuum of high-quality cancer care. Community engagement in research and intervention has been recognized as an important step in translating innovation to patients from diverse settings.^34^ CoC-accredited hospitals are required to report on the policies and procedures governing their multidisciplinary cancer case conferences, as well as the elements of discussion for each case, which include options and eligibility for supportive care services.^35^ Through a regulated site review process, all CoC-accredited sites are routinely evaluated on their adherence with the ACS CoC Optimal Resources for Cancer Care quality care standards. These standards include, but are not limited to, identifying and addressing barriers to care, initiating community cancer prevention and screening events, measuring compliance with operative standards, screening for psychosocial distress, and development of survivorship programs.^35^ Among 4 nationally recognized cancer accreditations and designations, CoC accreditation is the only one to recommend undertaking community needs assessments.^36^ CoC accreditation recommendations for addressing barriers and increasing community engagement are fundamental to understanding, acknowledging, and responding to a community’s cancer needs to improve outcomes for underserved populations.

The ACS has had a longstanding history of establishing and maintaining accreditation programs based on standards of care and data-driven quality assessment and improvement. For instance, the ACS National Accreditation Program for Breast Centers accreditation has been associated with improved adherence to nationally recognized breast cancer quality standards.^37^ In addition to cancer care, the ACS maintains accreditation and verification programs to improve quality for multiple surgical specialties. The Metabolic Bariatric Surgery and Quality Improvement Program, which is managed by the ACS and the American Society for Metabolic and Bariatric Surgery, evaluates and accredits bariatric Centers of Excellence in the US and Canada in accordance with nationally recognized bariatric surgical standards.^38^ Studies have reported bariatric centers with accreditation were associated with lower mortality and morbidity compared with nonaccredited centers.^39^ Additionally, centralization of bariatric surgery to accredited centers was associated with improved access for Black Medicare patients.^40^

### Limitations

This study has several important limitations. First, data from the NPCR database rely on certified cancer registry staff and are subject to reporting and abstracting errors. Second, data on patient comorbidities, postoperative complications, hospital type or volume, and treatment dates, to discern delays in treatment, were not available. Prior studies have reported that CoC-accredited hospitals compared with non–CoC-accredited hospitals were more frequently in urban locations and had higher case volumes.^41^ However, the constituency of CoC-accredited hospitals has changed over time and there are ongoing efforts to support attainment of CoC accreditation in underserved areas, such as rural areas.^42,43^ Further studies evaluating contemporary characteristics of CoC-accredited and non–CoC-accredited hospitals are warranted. Third, conversion of NCI SEER Summary Stage to the American Joint Committee on Cancer Staging Manual, 8th edition, may be associated with misclassification of disease stage. Fourth, SES was reported as predefined categorical Census tract poverty levels. While this study found that after adjustment, categorical Census tract poverty level was not associated with the outcomes evaluated, it must be acknowledged that other measures of SES not reported by the NPCR database, such as continuous Census tract poverty level or social vulnerability, may have a larger influence. Fifth, race and ethnicity were abstracted from medical records as recorded by health care facilities and practitioners, which has been associated with underclassification compared with self-reported race and ethnicity data.^44,45^ Additionally, due to Hispanic origin misclassification in North Dakota and Wisconsin, this category may be underestimated for any Hispanic ethnicity groups and overestimated for any non-Hispanic ethnicity groups.^45^

## Conclusions

In this cohort study, Black patients with colon cancer were more likely to receive GCC and have lower mortality risk when treated at CoC-accredited hospitals compared with non–CoC-accredited hospitals. Increasing access to high-quality cancer care at CoC-accredited hospitals may reduce variations in cancer treatment and outcomes for underserved populations.

## Notes

Supplement 1. **eTable.** Multivariable Adjusted Odds Ratio for Receipt of Colon Cancer Lymphadenectomy and Chemotherapy Measures for Non-Hispanic Black Patients With Low Socioeconomic Status

Supplement 2.
Data Sharing Statement
