# Supplementary material for: Hospital Accreditation Status and Treatment Differences Among Black Patients With Colon Cancer
Source: JAMA Netw Open. 2024 Aug 21;7(8):e2429563. doi: 10.1001/jamanetworkopen.2024.29563 (PMC11339660; doi:10.1001/jamanetworkopen.2024.29563)
Supplement: Supplement 1. — eTable. Multivariable Adjusted Odds Ratio for Receipt of Colon Cancer Lymphadenectomy and Chemotherapy Measures for Non-Hispanic Black Patients With Low Socioeconomic Status [file jamanetwopen-e2429563-s001.pdf]

Supplementary Online Content

Chan K, Palis BE, Cotler, JH, et al. Hospital accreditation status and treatment differences among Black patients with colon cancer. *JAMA Netw Open.* 2024;7(8):e2429563. doi:10.1001/jamanetworkopen.2024.29563

eTable 1. Multivariable Adjusted Odds Ratio for Receipt of Colon Cancer Lymphadenectomy and Chemotherapy Measures for Non-Hispanic Black Patients With Low Socioeconomic Status

This supplementary material has been provided by the authors to give readers additional information about their work.

© 2024 Chan K et al. *JAMA Network Open*.

eTable 1. Multivariable adjusted odds ratio for receipt of colon cancer lymphadenectomy and chemotherapy measures for non-Hispanic Black patients with low socioeconomic status

Variable	Group	Receipt of adequate lymphadenectomy during surgery, AOR (95% CI)	*P* value	Receipt of chemotherapy for stage III disease, AOR (95% CI)	*P* value
Sex	Male	1 [Reference]	NA	1 [Reference]	NA
	Female	1.32 (1.13 – 1.54)	<0.001	1.20 (0.97 – 1.49)	0.10
Age, y	18-49	1 [Reference]	NA	1 [Reference]	NA
	50-59	0.93 (0.67 – 1.30)	0.40	0.63 (0.41 – 0.96)	0.03
	60-69	0.71 (0.52 – 0.99)	0.02	0.48 (0.31 – 0.72)	0.02
	70-79	0.63 (0.44 – 0.90)	0.03	0.32 (0.20 – 0.51)	<0.001
	≥80	0.59 (0.40 – 0.86)	0.01	NA	NA
Insurance type	Medicare	0.86 (0.66 – 1.12)	0.56	0.95 (0.67 – 1.36)	0.51
	Private	0.94 (0.72 – 1.22)	0.80	1.24 (0.87 – 1.75)	0.21
	Medicaid	1 [Reference]	NA	1 [Reference]	NA
	Uninsured	0.83 (0.54 – 1.26)	0.55	0.80 (0.47 – 1.34)	0.19
	Other government[a]	0.95 (0.49 – 1.82)	0.88	1.31 (0.54 – 3.21)	0.51
Rural or urban	Metropolitan	2.00 (1.67 – 2.39)	<0.001	0.99 (0.74 – 1.32)	0.77
	Urban	1 [Reference]	NA	1 [Reference]	NA
	Rural	1.09 (0.67 – 1.77)	0.27	0.87 (0.41 – 1.84)	0.11
Hospital accreditation	Non-CoC-accredited	1 [Reference]	NA	NA	NA
	CoC-accredited	1.95 (1.67 – 2.29)	<0.001	2.23 (1.77 – 2.80)	<0.001
Disease stage[b]	Localized only	1 [Reference]	NA	NA	NA
	Regional by direct extension only	1.69 (1.38 – 2.06)	0.05	NA	NA
	Regional lymph node involvement	1.94 (1.63 – 2.30)	<0.001	NA	NA
Primary site	Right colon	1 [Reference]	NA	1 [Reference]	NA
	Transverse colon	0.48 (0.38 – 0.61)	0.01	0.90 (0.63 – 1.29)	0.85
	Left colon	0.42 (0.36 – 0.50)	<0.001	0.85 (0.67 – 1.07)	0.78
	Overlapping lesion of colon and colon, not otherwise specified	0.69 (0.43 – 1.11)	0.48	0.76 (0.43 – 1.37)	0.55

Abbreviations: AOR, adjusted odds ratio; CoC, Commission on Cancer.
[a] Over government insurance included TRICARE, military, Veterans Affairs, Indian Health Service
[b] Only patients with stage III colon cancer were eligible for chemotherapy

© 2024 Chan K et al. *JAMA Network Open*.
