# Supplementary material for: Hospital Accreditation Status and Treatment Differences Among Black Patients With Colon Cancer
Source: JAMA Netw Open. 2024 Aug 21;7(8):e2429563. doi: 10.1001/jamanetworkopen.2024.29563 (PMC11339660; doi:10.1001/jamanetworkopen.2024.29563)
Supplement: Supplement 2. — Data Sharing Statement [file jamanetwopen-e2429563-s002.pdf]

Data Sharing Statement

Chan. Hospital Accreditation Status and Treatment Differences Among Black Patients With Colon Cancer. *JAMA Netw Open*. Published August 21, 2024.
doi:10.1001/jamanetworkopen.2024.29563

Data
Data available: Yes
Data types: Deidentified participant data
How to access data: https://seer.cancer.gov/data/access.html
When available: With publication

Supporting Documents
Document types: None

Additional Information
Who can access the data: Researchers whose proposed use of the data has been approved
Types of analyses: Must submit request to https://seer.cancer.gov/data/access.html
Mechanisms of data availability: With signed data access agreement
